# Genetic variants and molecular profiling of 46,XY gonadal dysgenesis using whole-exome sequencing

**DOI:** 10.3389/fendo.2025.1560698

**Published:** 2025-04-11

**Authors:** Ning Zhang, Shuoming You, Jingjing Guo, Xingyu Chang, Junjun Qiu, Keqin Hua

**Affiliations:** ^1^ Department of Gynecology, Obstetrics and Gynecology Hospital, Fudan University, Shanghai, China; ^2^ Shanghai Key Laboratory of Female Reproductive Endocrine-Related Diseases, Shanghai, China; ^3^ Department of Gynecology, Obstetrics and Gynecology Hospital of Fudan University, Yangtze River Delta Integration Demonstration Zone (QingPu), Shanghai, China

**Keywords:** 46,XY gonadal dysgenesis, genetic etiology, whole exome sequencing, zinc finger, congenital heart disease

## Abstract

**Background:**

More than 60% of cases of 46,XY gonadal dysgenesis (GD), a condition classified as a disorder of sex development (DSD), remain unexplained, which is due to high genetic and clinical heterogeneity. Whole-exome sequencing (WES) is an efficient primary genetic diagnostic method; specifically, the use of WES in patients with 46,XY GD to explore the underlying genetic variants of the disorder may help us gain a deeper understanding of the pathogenesis and phenotype–genotype correlation of 46,XY GD.

**Methods:**

We performed WES and pedigree studies to investigate the underlying genetic etiology of patients with 46,XY GD (six patients and six familial controls). The variants were confirmed via Sanger sequencing, and detailed functional prediction of the discovered genetic variants was conducted. Furthermore, we performed *in-silico* protein structural analysis and protein thermodynamic analysis to further explore the pathogenicity of these variants. *GATA4* variants in patients with 46,XY GD with/without CHD and patients with cardiac disease alone were also analyzed.

**Results:**

We identified three novel pathogenic variants in *GATA4*:c.725G>C(p.Cys242Ser), *NR5A1*:c.370_380del(p.Pro124Glyfs*21), and *DHX37*:c.2020C>T(p.Arg674Trp), as well as one previously reported *MAP3K1*:c.1016G>A(p.Arg339Gln) variant. These variant sites are conserved among species and were predicted to be damaging according to functional algorithms and protein analyses. Additionally, 71.4% of the GATA4 amino acid changes in 46,XY GD were located in or close to the N-terminal zinc finger (N-ZF) domain. However, most *GATA4* pathogenic variants (31.82%) in patients with isolated cardiac diseases were located in transactivation domain 1 (TAD1), and only 9.09% of the variants were located in the N-ZF domain.

**Conclusion:**

The N-ZF domain may play an exclusive role in the mechanism of GATA4 in the pathogenesis of 46,XY GD; therefore, this domain may be an interesting topic for future investigation. This study enhances our understanding of the genetic etiology and pathogenesis of 46,XY GD, which may aid in the diagnosis and intervention of this disorder.

## 1 Introduction

46,XY gonadal dysgenesis (GD) is a congenital gonadal development disorder in individuals with a 46,XY karyotype that belongs to the spectrum of disorders/differences of sex development (DSD) (1). GD can be classified as either complete gonadal dysgenesis (CGD, also known as Swyer syndrome) or partial gonadal dysgenesis (PGD) (2). The characteristics of 46,XY CGD include female external genitalia, a well-developed Müllerian structure, and streak gonads without testicular differentiation, whereas 46,XY PGD is characterized by varying degrees of Wolffian (seminal vesicle, epididymis, and vas deferens) and Müllerian (uterus, fallopian tube, and upper third of the vagina) duct development, as well as virilization of external genitalia. The prevalence of 46,XY GD is approximately one per 8,000 births (3). An important characteristic of 46,XY GD is an increased lifetime risk of gonadal tumors, ranging from 15% to 35% (4). Early diagnosis is crucial to identify possible gonadal malignancies and excise ectopic gonads (if necessary), as well as to enable timely hormone treatments, obtain adequate peak bone mass, and provide timely psychosexual consultations. Genetic variants are the most important and common cause of 46,XY GD (5). An understanding of the genetic etiology of 46,XY GD is beneficial for pathogenesis studies, genetic counseling, clinical diagnosis, and treatment. Variants in several genes (including *GATA4*, *SRY*, *NR5A1*, *WNT4*, and *MAP3K1*, among others) are related to the pathogenesis of 46,XY GD (1). However, these variants explain only <40% of cases (6, 7).

Recently, whole-exome sequencing (WES) has become an important method for identifying new genes and loci related to genetic disease; currently, this method is a commonly used approach that exhibits a diagnostic rate of 35% (8). Therefore, we recruited six patients with 46,XY GD for this study. WES analysis was performed on these patients and their family members to explore genetic variants and phenotype–genotype correlations. Consequently, we discovered three novel pathogenic variants in *GATA4*, *NR5A1*, and *DHX37* and one previously reported *MAP3K1* variant in patients with 46,XY GD. The pathogenicity of these variants was demonstrated through *in-silico* studies. Combined with data from the published literature, the importance of the N-terminal zinc finger (N-ZF) domain of GATA4 in 46,XY GD was highlighted. Our research expands on the knowledge of the genetic mechanism underlying 46,XY GD.

## 2 Materials and methods

### 2.1 Patients

This study was approved by the Institutional Review Board Committee of the Obstetrics and Gynecology Hospital (2021–223), Fudan University (FE21192), and written informed consent was obtained from the participants and their parents. We recruited six patients who were diagnosed with 46,XY GD at the Obstetrics and Gynecology Hospital of Fudan University. Except for patient 3, blood samples and clinical data were collected from patients and their immediate family members (with the family members serving as six familial controls). Notably, patients 1 and 2, as well as patients 5 and 6, were identified as sibling sisters. The detailed clinical, laboratory, and molecular characteristics of the patients and their families were recorded.

### 2.2 Whole-exome sequencing

Genomic DNA was extracted from peripheral venous blood using a Blood Genome Column Medium Extraction Kit (9). The xGen Exome Research Panel v1.0 was used to analyze the genomic DNA and identify the exonic regions and flanking splice junctions of the genome (IDT, Coralville, IA, United States). The libraries were subsequently sequenced on a BGI T7 sequencer using PE150, with a minimum of 11 million reads. The paired-end reads were compared against the Ensembl GRCh37/hg19 human reference genome with the Burrows-Wheeler Aligner (BWA). Both single-nucleotide variants (SNVs) and small insertions/deletions (InDels) were identified using the Genomic Analysis Toolkit (GATK) program (10). The Exome Depth algorithm served as the basis for copy number variant (CNV) calling. The total read counts were mapped to each exon in the same batch as previously described (11). Variants were annotated using a Chigene online system that references 35 public databases. We categorized the candidate variants according to international standards established by the Sequence Variant Interpretation Working Group and the American College of Medical Genetics and Genomics (ACMG) (12).

### 2.3 Functional prediction of genetic results

The pathogenic effects of the identified variants were predicted *in silico* using four software algorithms: SIFT (http://provean.jcvi.org/index.php) (13), PolyPhen-2 (http://genetics.bwh.harvard.edu/pph2/) (14), MutationTaster (http://www.mutationtaster.org/) (15), and REVEL (https://sites.google.com/site/revelgenomics/) (16).

### 2.4 *In silico* protein structure analysis and protein thermodynamic analysis

*In-silico* protein structures were predicted for the *NR5A1*, *DHX37*, *MAP3K1*, and *GATA4* variants. First, AlphaFold2 (17) was used to predict the three-dimensional structures of the wild-type proteins encoded by these genes. Based on the predicted wild-type protein structures, we used SWISS-MODEL2 (18) to predict the structures of the variant proteins. The resulting crystal structures were visualized and annotated using PyMOL (http://www.pymol.org). LigPlot (19) was used to explore the hydrogen bond functions of the proteins at the two-dimensional level with and without alterations. Molecular docking analysis of the Zn atom in GATA4 was supplemented with AlphaFill (20). Kyte and Doolittle hydropathy plots were used to investigate the hydrophobicity of the protein region (https://fasta.bioch.virginia.edu/fasta_www2/fasta_www.cgi?rm=misc1&pgm=pkd).

Amino acid conservation between the different species was assessed using UniProt (https://www.uniprot.org), and disruption of the folding energy (ΔΔG) caused by missense variants in the domains was modeled using DUET (21), SDM (22), mCSM (23), and DeepDDG (24).

## 3 Results

### 3.1 Clinical and hormonal characteristics of patients with 46,XY GD and their families

Six patients diagnosed with 46,XY GD were included in this study. Patients 1 and 2 were diagnosed with PGD, whereas patients 3, 4, 5, and 6 were diagnosed with CGD. Notably, patients 1 and 2 were siblings, as were patients 5 and 6. The average age at diagnosis was 13.2 years. Basal hormonal evaluation results were available for all of the patients. Specifically, all of the patients exhibited increased gonadotropin levels, whereas patient 1 also had increased testosterone levels, which was consistent with the diagnosis of PGD. Patient 2 (the sister of patient 1) had lower testosterone levels, which may have been related to her young age and the fact that the gonads had not yet developed. None of the patients exhibited symptoms or signs of adrenal insufficiency, and normal basal adrenal function was observed in all of the patients. Moreover, all of the patients (except for patient 2) underwent bilateral gonadectomy; additionally, bilateral gonadoblastoma was observed in patient 3, who underwent three cycles of bleomycin–etoposide–cisplatin (BEP) chemotherapy. Notably, patients 1 and 2 also had congenital heart disease (CHD); specifically, patient 1 had a ventricular septal defect and a congenital diaphragm hernia, whereas patient 2 was diagnosed with a ventricular septal defect, an atrial septal defect, pulmonary arterial hypertension, and heart failure. The clinical features of these patients are presented in **Table 1**.

**Table 1:** Phenotypic descriptions, previous clinical findings, and genetic findings of the six patients.

Patient	1	2	3	4	5	6
**Diagnosis**	PGD	PGD	CGD	CGD	CGD	CGD
**CHD features**	VSD	VSD, ASD, PAH, heart failure	NA	NA	NA	NA
**Social gender**	Female	Female	Female	Female	Female	Female
**Age at presentation**	12 years	2 months	14 years	25 years	12 years	16 years
**Height (cm)**	163	NA	167	162	152	155
**Weight (kg)**	50	NA	55	53	48	60
**Menstruation**	Primary amenorrhea	/	Primary amenorrhea	Primary amenorrhea	Primary amenorrhea	Primary amenorrhea
**Secondary sexual development**	Absent	Absent	Absent	Absent	Absent	Absent
**Phenotype, external genitalia**	Female	Female	Female	Female	Female	Female
**Internal genitalia**	Fallopian tube-like structures and epididymis on each side, absent uterus and vagina	Cloacal malformation	Vagina present, infantile uterus (linear endometrium)	Absent uterus and vagina	Vagina present, primordial uterus	Vagina present, infantile uterus (linear endometrium)
**Age at gonad removal (years)**	12	–	14	26	12	16
**Gonadal position**	Inguinal canal	Inguinal canal	Pelvic cavity	Pelvic cavity	Pelvic cavity	Pelvic cavity
**Gonadal tumor**	–	–	Gonadoblastoma	–	–	–
**Gonadal histology**	Bilateral underdeveloped gonads with vas deferens and epididymis on both sides	–	Bilateral fibrous gonads with gonadoblastoma	Bilateral fibrous gonads	Bilateral streak gonads containing underdeveloped testis tissue, with fallopian tubes on both sides	Bilateral gonadal and testicular tissues with mature cystic teratoma
**Other clinical features**	Congenital diaphragm hernia	Atelectasis, emphysema, pleural insufficiency	–	Breast and vulva at Tanner stage 0	Clitoris approximately 2 cm in length	–
**LH (U/L)**	43.97	0.93	30.52	7.01	26.59	22.95
**FSH (U/L)**	119.3	39.25	83.69	31.62	111.67	54.98
**E2 (pmol/L)**	3	20	61.68	58.72	21	47.71
**T (nmol/L)^a^**	3.52	0.02	1.27	1.14	0.64	1.74

CGD, complete gonadal dysgenesis; PGD, partial gonadal dysgenesis; FSH, follicle-stimulating hormone; LH, luteinizing hormone; T, testosterone; VSD, ventricular septal defect; ASD, ventricular septal defect; PAH, pulmonary arterial hypertension.

^a^Reference range of T (female): 0.35–2.60 nmol/L.

### 3.2 WES analysis revealed four novel variants and one previously reported variant in five genes

**Figure 1:**
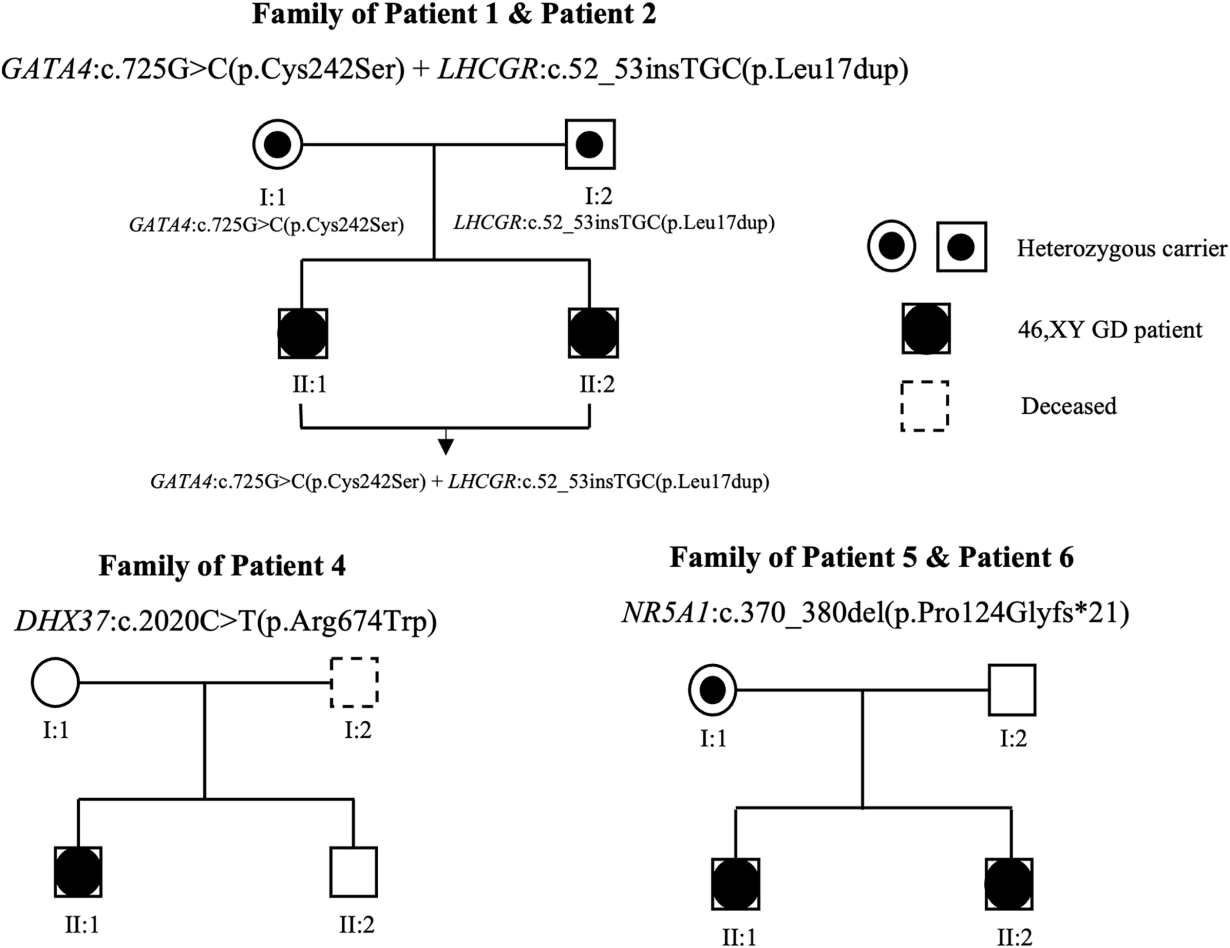
Family pedigrees associated with 46,XY GD. Genotypes are indicated on the chart. Squares and circles represent male and female family members, respectively. The black circles in the squares represent affected patients with 46,XY GD, who were raised as female. The symbols containing black dots represent heterozygous carriers.

We performed WES analysis on the patients and their family members (blood samples were available from the family members of all the patients except for patient 3) and identified four novel heterozygous variants and one previously reported heterozygous variant in the following five genes: *GATA4*, *LHCGR*, *MAP3K1*, *DHX37*, and *NR5A1* (detailed in **Table 2**). Variants of *GATA4*, *MAP3K1*, and *DHX37* were identified via Sanger sequencing (**Supplementary Figure S1**). Among these variants, the c.370_380del variant in *NR5A1* was a frameshift mutation, the c.52_c.53insTGC variant in *LHCGR* caused lysine duplication, and the remaining three variants were missense variants. None of the variants exhibited a minor allele frequency (MAF) (**Table 2**) in the dbSNP, 1000 Genomes, and gnomAD databases. Moreover, three variants exhibited a sex-specific, dominant inheritance pattern, which is consistent with the published literature (25). The family pedigrees are shown in **Figure 1**.

**Table 2:** Variants identified in patients with 46,XY GD and their frequencies in population databases.

Patient	Working diagnosis	Gene	Genomic position	cDNA position	AA change	Zygosity	Inheritance pattern	dbSNP	MAFs in population databases				
									1000 Genomes	1000 Genomes-South	1000 Genomes-North	gnomAD	gnomAD East Asian
1, 2	46,XY PGD	*GATA4*	chr8:11606533	c.725G>C	p.Cys242Ser	Heterozygous	Maternal	N	Absent	Absent	Absent	Absent	Absent
1, 2	46,XY PGD	*LHCGR*	chr2:48982773–48982774	c.52_c.53insTGC	p.Leu17dup	Heterozygous	Paternal	rs1553401008	Absent	Absent	Absent	Absent	Absent
3	46,XY CGD	*MAP3K1*	chr5:56160742	c.1016G>A	p.Arg339Gln	Heterozygous	Absent	rs1554034036	Absent	Absent	Absent	Absent	Absent
4	46,XY CGD	*DHX37*	chr12:125448965	c.2020C>T	p.Arg674Trp	Heterozygous	Absent	N	Absent	Absent	Absent	Absent	Absent
5, 6	46,XY CGD	*NR5A1*	chr9:127262859–127262869	c.370_380del	p.Pro124Glyfs*21	Heterozygous	Maternal	N	Absent	Absent	Absent	Absent	Absent

CGD, complete gonadal dysgenesis; PGD, partial gonadal dysgenesis; MAF, minor allelic frequency.

### 3.3 *In silico* functional analysis confirmed the pathogenicity of the missense variants

To obtain additional information about the possible functional consequences of the identified variants, we conducted *in-silico* conformational analysis. We analyzed the missense variants in the *GATA4*, *MAP3K1*, *DHX37*, and *LHCGR* genes via SIFT, PolyPhen-2, MutationTaster, and REVEL. Among the identified variants, the *GATA4*, *MAP3K1*, and *DHX37* variants were predicted to be damaging via all four algorithms, whereas the *LHCGR* variant was predicted to be polymorphic via MutationTaster (**Table 3**). According to the ACMG criteria (26), the *NR5A1* variant was classified as pathogenic, whereas the *GATA4*, *MAP3K1*, and *DHX37* variants were classified as variants of uncertain significance.

**Table 3:** *In-silico* prediction analysis of variants identified in patients with 46,XY GD.

Patient	Gene	Nucleotide change	AA change	*In-silico* prediction tools				ACMG criteria (2019)
				SIFT	PolyPhen-2	MutationTaster	REVEL	
1, 2	*GATA4*	c.725G>C	p.Cys242Ser	Damaging	Probably damaging	Disease-causing	Deleterious	VUS
1, 2	*LHCGR*	c.52_c.53insTGC	p.Leu17dup	–	–	Polymorphism	–	VUS
3	*MAP3K1*	c.1016G>A	p.Arg339Gln	Damaging	Probably damaging	Disease-causing	Deleterious	VUS
4	*DHX37*	c.2020C>T	p.Arg674Trp	Damaging	Probably damaging	Disease-causing	Deleterious	VUS
5, 6	*NR5A1*	c.370_380del	p.Pro124Glyfs*21	–	–	–	–	LP

SIFT, Sorting Intolerant from Tolerant; VUS, variant of uncertain significance; LP, likely pathogenic.

### 3.4 Protein conformational changes caused by the variants

**Figure 2:**
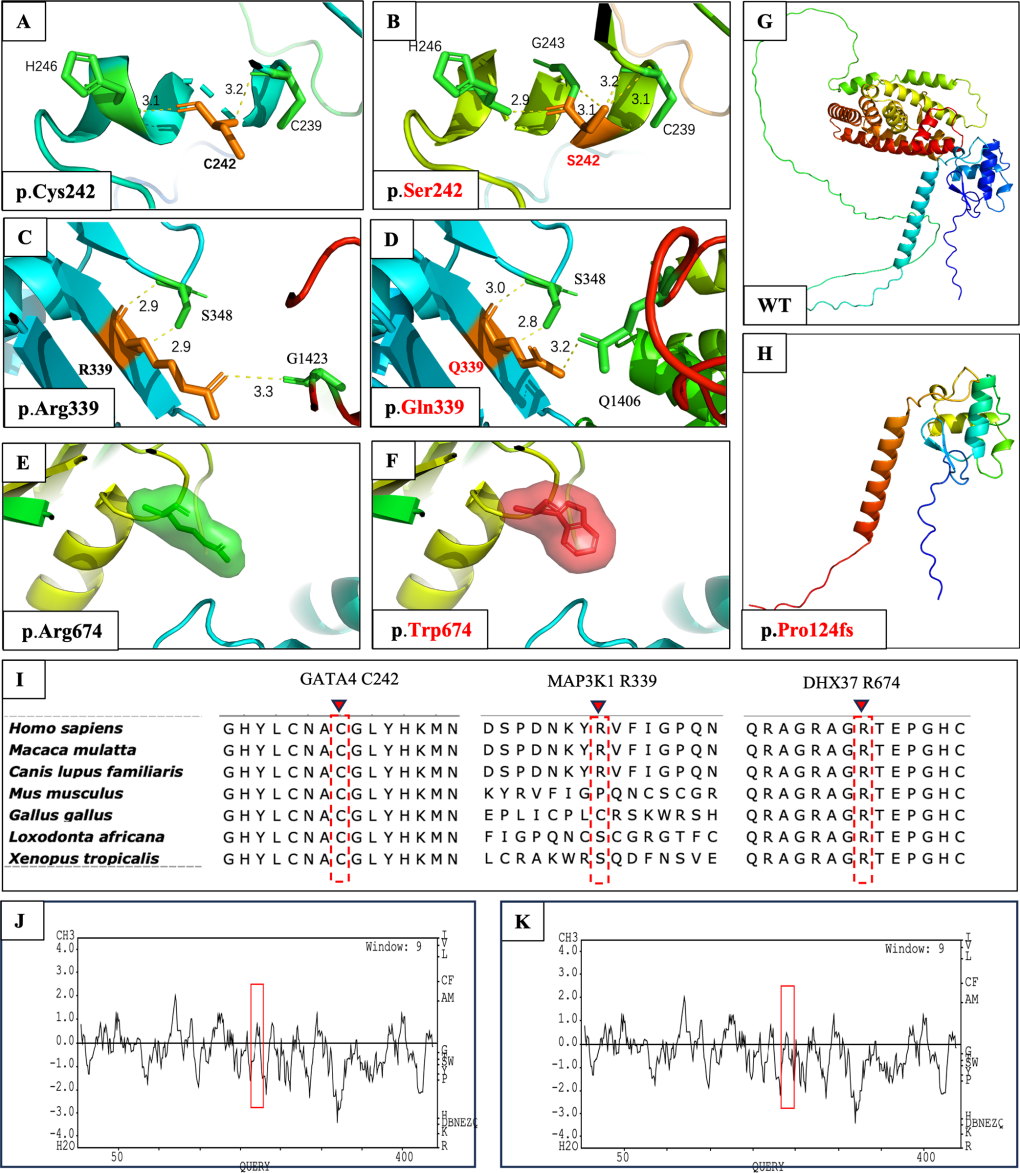
*In-silico* 3D protein structural prediction of *GATA4*, *MAP3K1*, *DHX37*, and *NR5A1* variants using AlphaFold2 and SWISS-MODEL2. The amino acid residue is marked in orange, and interacting residues are marked in green. Hydrogen bonds are depicted as yellow dashed lines, with the bond length values shown in **(E–H)**. **(A)** Close-up view of wild-type Cys242 in GATA4. Cys242 is predicted to form a 3.1-Å hydrogen bond with His246 and a 3.2-Å hydrogen bond with Cys239. **(B)** The Ser242 amino acid change is predicted to influence the original hydrogen bond with Cys239 and form a new hydrogen bond with Gly243. **(C)** Close-up view of wild-type Arg339 in MAP3K1. This residue is predicted to form two 2.9-Å hydrogen bonds with Ser348 and one 3.3-Å hydrogen bond with Gly1423. **(D)** In the MAP3K1 amino acid change, the previously mentioned hydrogen bond with Ser348 and Gly1423 was changed. Moreover, a new hydrogen bond was formed with Arg1406. **(E)** Close-up view of wild-type Arg674 in DHX37 and its interaction with peripheral domains. The residue of interest is depicted as a green sphere. **(F)** The Trp674 amino acid change is depicted as a red sphere. No evident interactional or structural changes were observed before or after the alteration. **(G)** Wild-type protein structure of NR5A1. **(H)** Protein structure of NR5A1:p.Pro124Glyfs*21. The alteration causes the loss of a large fragment of the protein. **(I)** Multiple alignments of parts of the GATA4, MAP3K1, and DHX37 protein sequences across various species. The locations of the newly identified human amino acid change are shown as dotted red rectangles with inverted red triangles at the top. Kyte and Doolittle hydropathy plots of the GATA4 protein **(J)** before and **(K)** after the p.Cys242Ser alteration. A score >0 indicates hydrophobicity, and a score <0 indicates hydrophilicity. Lower positive values indicate greater hydrophilicity. The alteration caused increased hydrophilicity in the region between codons 230 and 250, as outlined by the red boxes.

To obtain additional information about the possible functional consequences of the identified variants, we conducted *in-silico* conformational analysis on the variants according to at least one of the following conditions: 1) predicted to be pathogenic according to the ACMG criteria or 2) predicted to be damaging or disease-causing by three out of four algorithms (SIFT, PolyPhen-2, MutationTaster, and REVEL). The predicted 3D protein structures are shown in **Figure 2**.

In wild-type GATA4, the cysteine at position 242 was predicted to form a 3.1-Å hydrogen bond with the histidine at position 246 and a 3.2-Å bond with the cysteine at position 239 (**Figure 2A**). In patient 1, cysteine 242 was replaced by serine, which was predicted to form a new dihydrogen bond structure with Cys 239 (3.1 Å and 3.2 Å) by 3D and 2D modeling (**Figure 2B**; **Supplementary Figures S2A, B**). Due to the fact that serine is a hydrophilic polar amino acid, we also investigated the hydrophilicity of the protein region via Kyte and Doolittle hydropathy plots. We observed that the p.Cys242Ser amino acid change caused increased hydrophilicity in the region between codons 230 and 250 (**Figures 2J, K**). Furthermore, the ΔΔG results confirmed the pathogenicity of the amino acid change (**Supplementary Table S1**).

In wild-type MAP3K1, the arginine at position 339 was predicted to form two 2.9-Å hydrogen bonds with the serine at position 348 and one 3.3-Å hydrogen bond with the glycine at position 1423 (**Figure 2C**). In patient 3, the arginine residue was replaced with glutamine, which was predicted to interfere with the dihydrogen bond structure with serine 348 (3.0 Å and 2.8 Å), thereby causing a loss of the hydrogen bond with glycine 1423 and the formation of a new hydrogen bond with glutamine 1406 (3.2 Å) (**Figure 2D**). Combined with the 2D modeling (**Supplementary Figures S2C, D**) and ΔΔG results (**Supplementary Table S1**), these findings indicated possibly damaging structural or functional consequences of this amino acid change.

Compared with the wild-type protein, the DHX37 amino acid change, which results in tryptophan being located at position 674, was not predicted to cause any evident structural or functional changes in the 3D structure (**Figures 2E, F**). However, 2D modeling predicted that this change resulted in the loss of dihydrogen bonds with residues 670 (2.74 Å and 3.35 Å) and 277 (2.69 Å and 3.11 Å), as well as a loss of a hydrogen bond with residue 436 (2.92 Å) (**Supplementary Figures S2E, F**). The loss of these hydrogen bonds could cause conformational instability in the local domain, which was supported by the ΔΔG results (**Supplementary Table S1**).

The 3D structural change caused by the in-frame deletion from position 370 to position 380 in steroidogenic factor-1 (SF-1) is shown in **Figure 2H**. This 10-residue deletion caused codon 144 in the mRNA to become a stop codon, which resulted in the loss of a large fragment of the protein and most likely disrupted protein function.

### 3.5 Decreased Zn-binding efficiency due to GATA4 alterations was predicted and may play an important role in patients with 46,XY GD

**Figure 3:**
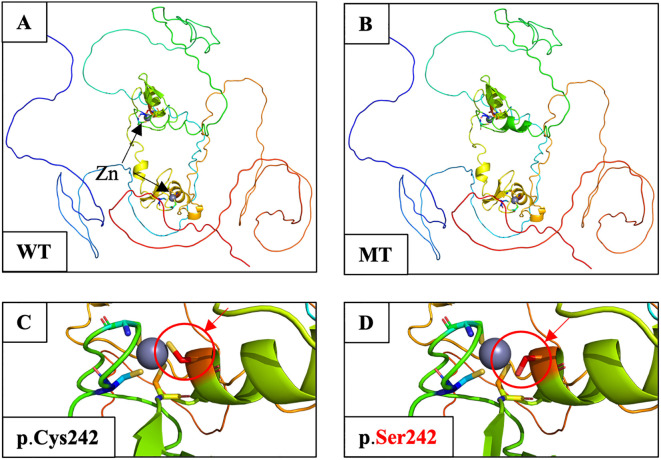
Molecular docking analysis of Zn atoms in wild-type and mutant GATA4 using AlphaFill. **(A)** Structure of WT GATA4 showing the Zn coordination and binding of DNA. Two Zn atoms are indicated by black arrows. **(B)** The p.Cys242Ser amino acid change was predicted to disrupt Zn binding and protein stability. **(C)** Close-up image of the Zn-binding site in wild-type GATA4. The Zn-binding site is marked by a red circle and an arrow. **(D)** Close-up of the p.Cys242Ser amino acid change showing the reduced Zn-binding site with the protein domain (shown by a red circle and arrow). The alteration was predicted to decrease the binding efficacy, which would affect the normal biochemical function of the protein.

**Figure 4:**
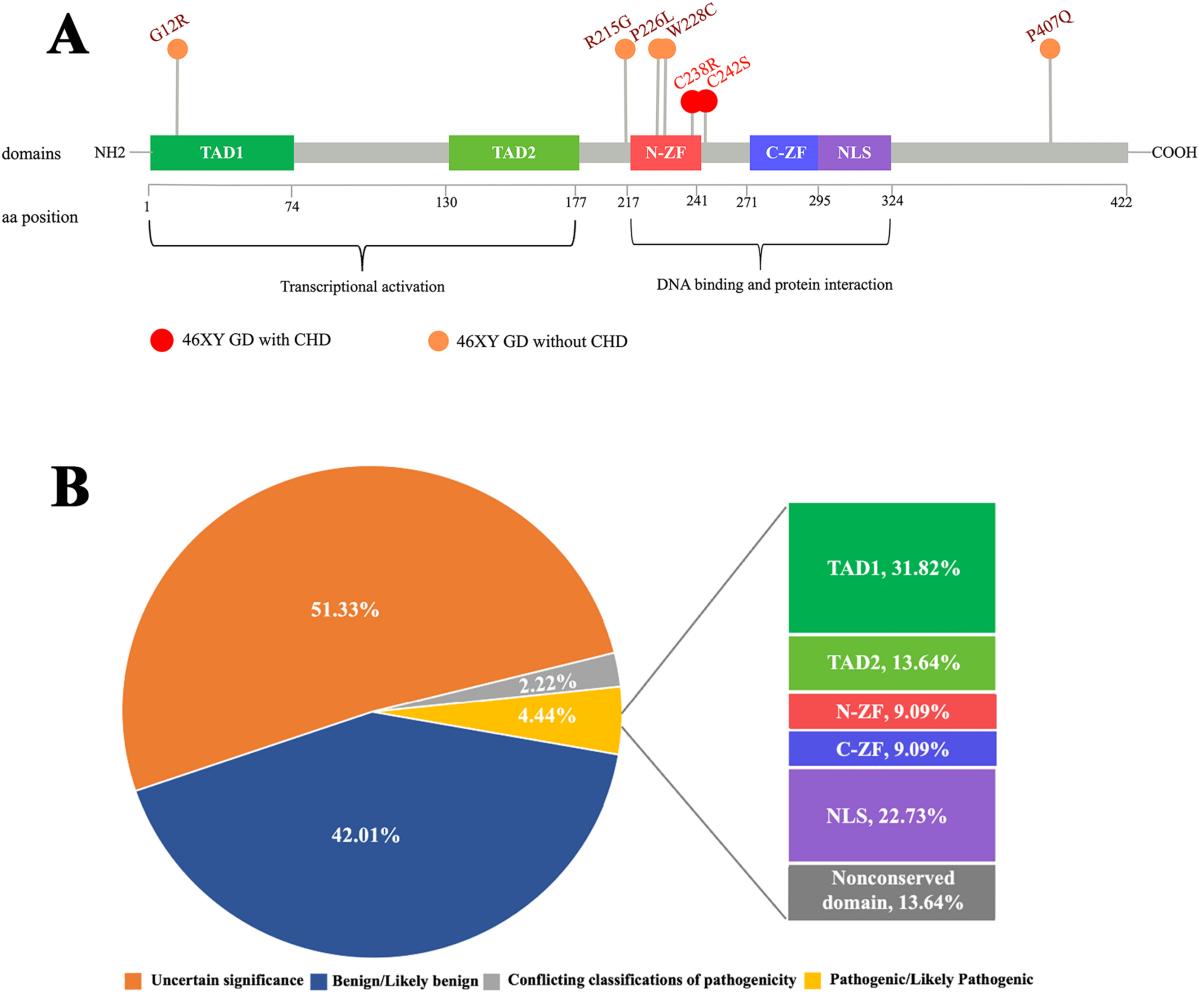
Analysis of the examined GATA4 amino acid change. **(A)** Schemes of the structure of the GATA4 protein and location of GATA4 amino acid changes in patients with 46,XY GD, as well as other patients with 46,XY GD with/without CHD. GATA4 contains two distinct zinc finger domains (N-ZF and C-ZF) and a C-terminal nuclear localization sequence (NLS), which consists of a DNA-binding domain and a protein–protein interaction domain. Transcriptional activation domains (TADs) are located in the N-terminus. Most GATA4 amino acid changes in patients with 46,XY GD with/without CHD were concentrated in the N-ZF domain. **(B)** Analysis of the germline classification of *GATA4* variants in patients with cardiac diseases alone and the location of pathogenic/likely pathogenic *GATA4* variants, as shown in the data from ClinVar.

As a Zn transcription factor, the Zn-binding domain of GATA4 is essential for its role in both types of gonadal development. Therefore, we performed a molecular docking analysis of Zn with the GATA4 amino acid changes of patients 1 and 2. We discovered that the GATA4 amino acid change site was located in the Zn-binding zone. This amino acid change decreased the number of Zn-binding sites within the protein domain, which reduced the strength and efficiency of binding and impacted normal biochemical function (**Figure 3**). This decreased Zn-binding efficiency was also indicated via 2D structure modeling (**Supplementary Figure S2B**). To further explore the role of different domains of GATA4 in 46,XY GD and CHD, we summarized the locations of *GATA4* variants in patients diagnosed with 46,XY GD with/without CHD from the published literature. We found that 71.4% of the *GATA4* variants in 46,XY GD were located in/close to the N-ZF domain (**Table 4**, **Figure 4A**). Additionally, we analyzed the data of *GATA4* variants associated with isolated cardiac diseases from ClinVar (https://www.ncbi.nlm.nih.gov/clinvar/) and determined that only 9.09% of pathogenic/likely pathogenic variants were found to be located in the N-ZF domain; moreover, most of the variants were located in transactivation domain 1 (TAD1, 31.82%), followed by nuclear localization signal (NLS, 22.73%), transactivation domain 2 (TAD2, 13.64%), no conserved domain (13.64%), and N-ZF (9.09%) (**Figure 4B**). These results revealed that different domains of GATA4 play different roles and the N-ZF domain of GATA4 plays an important role for 46,XY GD.

**Table 4:** GATA4 variants in 46,XY GD patients with/without CHD.

Disease	CHD	Variant position	Nucleotide change	AA change	Inheritance pattern	Zygosity	Other DSD genes	References
46,XY GD	VSD	Close to N-ZF	c.725G>C	p.Cys242Ser	Maternal	Heterozygous	*LHCGR*: (c.52_c.53insTGC(p.L17dup))	Our patients (patients 1 and 2)
46,XY GD	Complexed CHD^a^	N-ZF	c.712T>C	p.Cys238Arg	*De novo*	Heterozygous	NA	Martinez de LaPiscina et al. (27)
46,XY GD	NA	Close to N-ZF	c.643A>G	p.Arg215Gyl	NA	Heterozygous	NA	Choi et al. (28), Igarashi et al. (29)
46,XY GD	NA	N-ZF	c.677C>T	p.Pro226Leu	Maternal	Heterozygous	*LHCGR*:c.1660C>T(p.Arg554Stop)	Martinez de LaPiscina et al. (27)
46,XY GD	NA	N-ZF	c.684G>C	p.Trp228Cys	NA	Heterozygous	*LRP4*:c.5660C>G(p.Ser1887Cys)	Martinez de LaPiscina et al. (27)
46,XY GD	NA	TAD1	c.34G>C	p.Gly12Arg	Maternal	Heterozygous	NA	Globa et al. (30)
46,XY GD	NA	Non-conserved domain	c.1220C>A	p.Pro407Gln	NA	Heterozygous	Hemizygous for *AR*:c.226C>T(p.Q76*) (one out of five patients)	Choi et al. (28), Igarashi et al. (29)

GD, gonadal dysgenesis; N-ZF, N-terminal zinc finger; CHD, congenital heart disease; VSD, ventricular septal defect; TAD1, transcription activation domain 1; NA, not available.

^a^Ventricular septal defect, congenital compression of the left bronchus leading to an asymmetry in the caliber of the pulmonary branches with a hypoplastic left branch.

## 4 Discussion

An understanding of the genetic etiology of 46,XY GD is beneficial for pathogenesis studies, genetic counseling, clinical diagnosis, and treatment. However, the genetic etiology for most patients with 46,XY GD, as well as the exact genetic mechanism and phenotype–genotype correlation, remains unknown. In this study, we identified three novel pathogenic variants, namely, *GATA4*, *NR5A1*, and *DHX37* variants, as well as one previously reported *MAP3K1* variant, in six patients with 46,XY GD by WES. All of these variants were predicted to be damaging using both functional algorithms and protein modeling. To further explore the *GATA4* variants involved in 46,XY GD with/without CHD and their possible phenotype–genotype correlation, we performed molecular docking analysis of Zn in the *GATA4* variants. Furthermore, we summarized the locations of *GATA4* variants in patients with 46,XY GD with/without CHD and in patients with cardiac disease alone.

*GATA4* is expressed in the somatic cells of both mice and humans. This gene has been demonstrated to play important roles in transcriptional activity (31). *GATA4* variants can interfere with the transcriptional activation of gonadal promoters such as *NR5A1*, thereby potentially affecting Sertoli cell development in the testis and causing 46,XY DSD (32). In our study, patients 1 and 2 [who were sisters bearing the novel pathogenic *GATA4* variant known as the c.725G>C(p.Cys242Ser) variant] were both diagnosed with 46,XY PGD. This novel missense c.725G>C variant in *GATA4* has been shown to occur at a site that is highly conserved among species.GATA4 proteins have several conserved domains, including an N-ZF domain, a C-terminal zinc finger domain (C-ZF), TAD1, TAD2, and NLS. Our *GATA4* variant was observed to be located in the N-ZF domain. Notably, 71.4% of the *GATA4* variants in 46,XY GD were located in or close to the N-ZF domain, which strengthens the importance of this area in sex development. This domain plays an essential role in protein stability and is necessary for the protein to interact with other transcription factors (27). In our study, the *in-silico* analysis revealed that the p.Cys242Ser amino acid change of GATA4 in the N-ZF domain decreased the Zn-binding efficacy. This change may affect interactions with important factors involved in gonadal development, such as NR5A1, FOG2, and AMH, thereby leading to the 46,XY GD phenotype (33). Based on these findings, we suggest the implementation of clinical recommendations to prioritize screening for variants in the N-ZF domain of the GATA4 among individuals with 46,XY GD.

GATA4 is not only important for gonadal development but also well known for its role in cardiac development. *GATA4* variants can disrupt the formation of the cardiac tube, separation and development of the outflow tract, formation of the atrioventricular canal, and development of the cardiac conduction system, which can contribute to CHD (34). Intriguingly, patients 1 and 2 with the *GATA4* variant also demonstrated co-occurrence with CHD. Similarly, Martinez de LaPiscina et al. (27) reported a p.Cys238Arg variant in *GATA4* in a patient with 46,XY GD with complex CHD, which was also located in the N-ZF domain. In humans, more than 120 *GATA4* variants have been found to be associated with cardiac disease, whereas only 14 variants have been observed to be related to 46,XY DSD (27), thereby suggesting that *GATA4* variants are more likely to result in CHD than DSD; however, the underlying reason for this effect remains unclear. A possible explanation is that some *GATA4* variants retain partial DNA-binding activity, thus exhibiting different degrees of transcriptional activation of gonadal promoters, such as *NR5A1* (28).

Furthermore, we analyzed the data concerning *GATA4* variants in patients with cardiac diseases alone, which were obtained from ClinVar. Unlike the *GATA4* variant loci identified in patients with 46,XY GD, only 9.09% of the *GATA4* variants in patients with cardiac diseases alone were located in the N-ZF domain; moreover, most of the variants (31.82%) were located in TAD1. These results underscore the critical importance of the N-ZF domain in cases of 46,XY GD from a different perspective. Interestingly, some patients with 46,XY GD possessing *GATA4* variants also exhibited the CHD phenotype, whereas others did not exhibit this phenotype, and the underlying mechanism for this difference is still unknown. These findings indicate that little is known about the specific underlying genetic mechanisms and phenotype–genotype correlations of 46,XY GD and CHD (28), which warrant further explorations.

We also identified two novel heterozygous variants in *DHX37* and *NR5A1*. Pathogenic variants in *DHX37* and *NR5A1* are prevalent causes of 46,XY GD and explain approximately 11%–20% of cases (2, 35–37). *DHX37* is a newly reported 46,XY GD-related gene that plays critical roles in both early human testis determination and the maintenance of testicular tissue during the early phase of testis development (7, 38, 39). The novel c.2020C>T variant detected in patient 4 is located in the VI region of the functional RecA2 domain near the C-terminus; additionally, this site is highly conserved among various species, including yeast, and is involved in ATP binding, hydrolysis, and cross-talk with the unwinding machinery (38). *NR5A1*, which encodes SF-1, is a key gene involved in testis differentiation (40). We identified a novel c.370_380del variant in the DNA-binding domain of *NR5A1* that caused a frameshift variant. This variant has not been previously reported; additionally, it was classified as strongly pathogenic (PVS1-null variant) according to the ACMG criteria (26) and was predicted via 3D modeling to cause the loss of a large fragment of the protein. This maternally inherited variant was also detected in the patient’s older sister (patient 6), who was also diagnosed with 46,XY GD, whereas the patient’s mother had a normal phenotype, which was consistent with a sex-limited, dominant pattern of inheritance. Notably, the risk of germ cell tumors related to *NR5A1* variants may be associated with the severity of the phenotype and variant type (41), and *DHX37* variants may increase cancer risk when considering their function in ribosomal biogenesis (42), which warrants further investigation. Therefore, cancer screening may be needed for patients with 46,XY GD who harbor *DHX37* or *NR5A1* variants. Finally, we also identified one previously reported variant in *MAP3K1*, which plays an important role in gonadal sex determination. Specific variants in *MAP3K1* cause domination of the female sex-determining pathway, and variants have been observed in 15%–20% of sporadic and familial cases of 46,XY DSD (43). The variant that was observed in our study is located in the SWI2/SNF2 and MuDR (SWIM) domains (residues Glu303 to His393) (44) and may affect the interaction between *MAP3K1* and c-Jun, which is consistent with previous reports (45).

In summary, via WES in six patients with 46,XY GD, we identified three novel pathogenic variants in *GATA4*, *NR5A1*, and *DHX37* and one previously reported pathogenic variant in *MAP3K1*. All of these variants are conserved among various species and were predicted to be pathogenic by functional algorithms and *in-silico* protein analyses. We also discovered that the N-ZF domain may play an exclusive role in the mechanism of GATA4 in 46,XY GD patients. These results advance our understanding of the genetic etiology and phenotypical variability of 46,XY GD, which may improve diagnosis and intervention therapies. However, in conjunction with previous studies, the currently reported variants can only explain a limited number of cases of 46,XY GD. More genetic and functional studies need to be performed in larger samples of patients with 46,XY GD in the future.

## Data Availability

The data presented in the study are deposited in the NCBI repository https://www.ncbi.nlm.nih.gov/sra, accession number: PRJNA1243706.
