# Supplementary material for: Genetic variants and molecular profiling of 46,XY gonadal dysgenesis using whole-exome sequencing
Source: Front Endocrinol (Lausanne). 2025 Apr 11;16:1560698. doi: 10.3389/fendo.2025.1560698 (PMC12021639; doi:10.3389/fendo.2025.1560698)
Supplement: Supplementary file 1 [file DataSheet1.docx]

**Supplementary materials**:


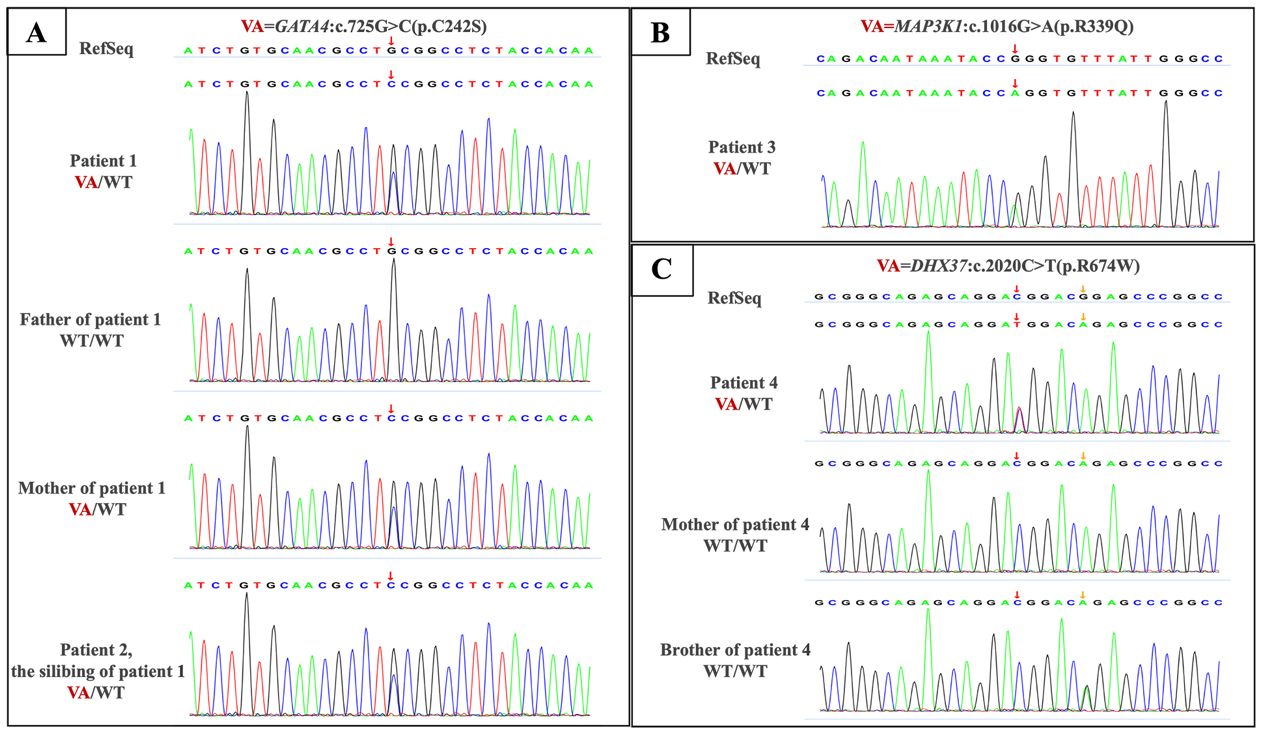


**Figure S1. Verification of variants identified in 46,XY GD patients by Sanger sequencing**. A. The verification of *GATA4*:c.725G>C(p.Cys242Ser) variant. B. The verification of *MAP3K1*:c.1016G>A(p.Arg339Gln) variant. C. The verification of *DHX37*:c.2020C>T(p.Arg674Trp) variant. Abbreviations:VA:variant;WT:wild type.


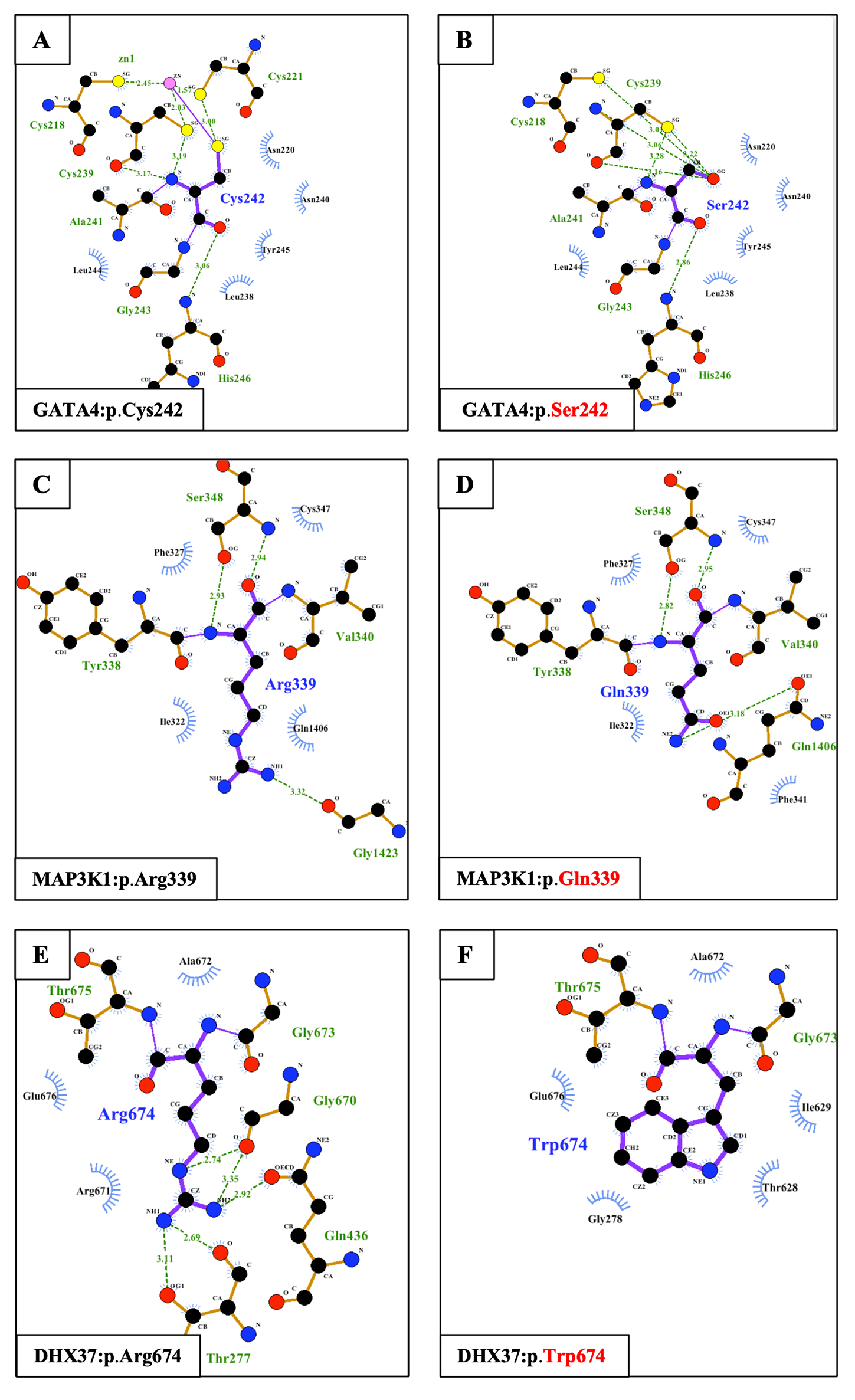


**Figure S2. Two-dimension intermolecular analysis of hydrogen bond function at the position of the variants using Uniprot**. Hydrogen bonds are showed in dashed green line, and protein residues which form non-bonded interaction with residues of interest are marked by blue bent spokes. **(A)** The intermolecular analysis of wildtype GATA4. **(B)** The mutant residue is predicted to loss bond with Zn atom and change the hydrogen bonds with nearby residue. **(C)**The intermolecular analysis of wildtype MAP3K1. The wildtype Arg339 form two hydrogen bonds with Ser348(2.93Å, 2.94Å), and one hydrogen bond with Gly1423(3.32Å). **(D)** The mutant Gln339 has changed bihydrogen structure with Ser348(2.95Å、2.82Å). Moreover, the mutant Gln339 loss the hydrogen bond with Gly1423 and form a 3.18Å hydrogen bond with Gln1406. This result is in consistent with 3D protein structural analysis result**. (E)** The intermolecular analysis of wildtype DHX37, Arg674 forms bihydrogen bonds with residue at position 670(2.74Å, 3.35Å) and 277(2.69Å, 3.11Å) and one hydrogen bond (2.92Å) with residue at position 436. **(F)**After alteration, Trp674 fails to form hydrogen bond with nearby amino acids. This could lead to local structural change and affect stability of the protein. These changes could influence protein structure and harm its function.

**Table S1. Predicted ∆∆G of amino acid changes**

| Gene | Mut | DUET^1^ | SDM^2^ | mCSM^3^ | DeepDDG^4^ | predicted result |
| --- | --- | --- | --- | --- | --- | --- |
| *GATA4* | C242S | -1.98 | -1.4 | -2.017 | -2.129 | unstable |
| *MAP3K1* | R339Q | -0.381 | -0.92 | -0.343 | -0.831 | unstable |
| *DHX37* | R674W | -0.9 | 0.08 | -1.057 | -0.93 | unstable |

1/2/3/4: positive value indicates the mutant type is more stable, while negative value indicates the mutant type is more unstable.
